# Translational regulation of replication stress responses

**DOI:** 10.1093/plcell/koad150

**Published:** 2023-05-23

**Authors:** Louis-Valentin Méteignier

**Affiliations:** Assistant Features Editor, The Plant Cell, American Society of Plant Biologists, USA; PHIM Plant Health Institute, Univ Montpellier, INRAE, CIRAD, Institut Agro, IRD, Montpellier 34398, 7 France

DNA replication faithfully copies the genetic information required for cell division and differentiation. In specific biological contexts, DNA replication may be impaired, and this can decrease plant growth and yield. The ATAXIA TELANGIECTASIA RAD3-related (ATR) kinase senses replication errors and induces a stress response that resolves errors that otherwise might lead to cell death (Pedroza-Garcia et al. 2021). Together with ATR, the WEE1 kinase and the SUPPRESSOR OF GAMMA-response 1 (SOG1) transcription factor are master activators of replication stress responses in plants (Bourbousse et al. 2018; Nisa et al. 2019). However, how the ATR-WEE1-SOG1 axis is regulated remains poorly understood.

In this issue of *The Plant Cell*, **Chen et al. (2023)** propose that translational derepression of *SOG1* mRNAs is an important step of ATR-WEE1–mediated replication stress responses (see Fig.). The authors initiated their study by performing a genetic screen in Arabidopsis to identify suppressors of the replication stress hypersensitivity phenotype of an *atr* mutant. One of the suppressors identified encodes for GENERAL CONTROL NONDEREPRESSIBLE 20 (GCN20), which is part of a protein complex that inhibits translation (Izquierdo et al. 2018). Introducing a *gcn20* mutation in *atr* or *wee1* mutant plants suppressed the replication stress hypersensitivity of both mutants, suggesting that ATR and WEE1 negatively regulate GCN20. To investigate how this negative regulation could occur, the authors tested if WEE1 and GCN20 interact, which was confirmed using yeast 2-hybrid, split luciferase, co-immunoprecipitation, and in vitro pull-down assays.

**Figure.**
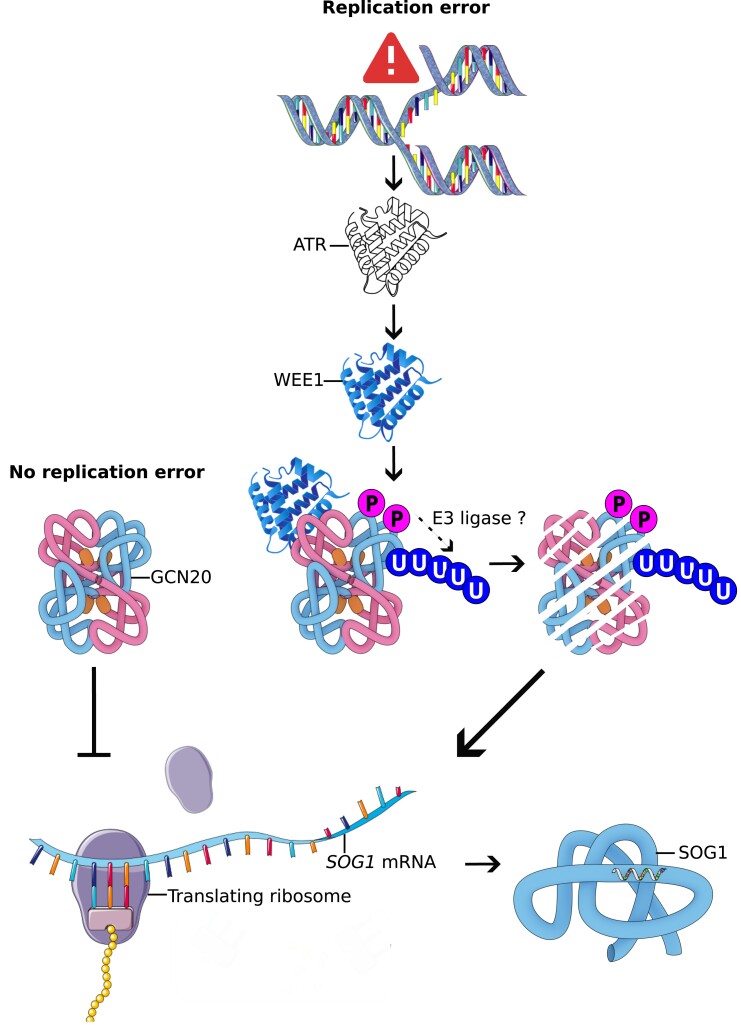
Replication stress responses are controlled at the translational level. Upon sensing replication stress, ATR signals through WEE1 that phosphorylates GCN20. Phosphorylated GCN20 is then targeted for polyubiquitination and degradation by an unknown E3 ubiquitin ligase, which allows for the translation of *SOG1*. Adapted from Chen et al. (2023). Images were taken from bioicons.com.

The authors hypothesized that WEE1 kinase activity could be involved in regulating GCN20. By using a phosphorylation-dependent protein gel shift assay, the authors observed that GCN20 migrated at a higher molecular weight when an active WEE1 kinase was co-incubated in vitro. Furthermore, they observed that GCN20 migrated more slowly when overexpressed in wild-type (WT) protoplasts compared to *wee1* mutant protoplasts and only in conditions resulting in replication stress. By mass spectrometry, the researchers identified 2 phosphorylated amino acid residues in GCN20 that they used to design nonphosphorylatable and phosphomimetic forms of GCN20. They observed that mutation of only 1 residue to a nonphosphorylatable residue partially suppressed the WEE1-dependent band shift of GCN20, whereas the double mutation abolished it, confirming the 2 residues as bona fide WEE1 targets. Based on previously published work, the authors noted that protein phosphorylation events associated with negative regulation are often correlated with polyubiquitination. Immunoprecipitation and immunoblotting of GCN20 to test this model showed that polyubiquitination levels were higher in WT plants than in *wee1* mutants in conditions resulting in replication stress. Consistent with a negative effect of WEE1 on GCN20, recombinant GCN20 degraded faster in protein extracts from WT plants compared to *wee1* mutants. Importantly, GCN20 degraded faster in protoplasts expressing WEE1 as opposed to the kinase-dead WEE1. The polyubiquitination and degradation levels of the phosphomimetic GCN20 were higher than that of the non-phosphorylatable variant, reinforcing the notion that WEE1-dependent phosphorylated residues induce GCN20 degradation through polyubiquitination.

Knowing that GCN20 represses the translation of the functional analog of *SOG1*, *p53*, in human cells, the authors hypothesized that Arabidopsis GCN20 could translationally regulate *SOG1* mRNAs in a similar way. They therefore analyzed the global polysome profile and polysome loading of *SOG1*. The global level of polysomes increased in the *gcn20* mutant and decreased in GCN20-overexpressing plants, as expected if GCN20 is a global translational repressor in plants. Consistent with that observation, *SOG1* mRNA loading in polysomes declined when GCN20 was overexpressed, whereas it increased in the *gcn20* mutant.

The authors proposed a model where, in the absence of replication stress, the ATR-WEE1 module is not active, allowing for the accumulation of GCN20 and therefore *SOG1* translational inhibition. Upon sensing replication stress, ATR signals through WEE1, which then phosphorylates GCN20. The phosphorylated form of GCN20 is targeted to the proteasome for degradation, thereby allowing the translation of *SOG1* (see Fig.) to mediate replication stress responses. Future work could aim at the identification of the E3 ubiquitin ligase involved in the phosphorylation-dependent degradation of GCN20.
